# Diversity of neurovascular coupling dynamics along vascular arbors in layer II/III somatosensory cortex

**DOI:** 10.1038/s42003-021-02382-w

**Published:** 2021-07-09

**Authors:** Ravi L. Rungta, Marc Zuend, Ali-Kemal Aydin, Éric Martineau, Davide Boido, Bruno Weber, Serge Charpak

**Affiliations:** 1 INSERM U1128, Laboratory of Neurophysiology and New Microscopy, Université Paris Descartes, Paris, France; 2 Faculté de Médecine Dentaire, Université de Montréal, Montréal, QC Canada; 3 Centre Interdisciplinaire de Recherche sur le Cerveau et l’Apprentissage, Université de Montréal, Montréal, QC Canada; 4 Groupe de Recherche sur le Système Nerveux Central, Université de Montréal, Montréal, QC Canada; 5 Institute of Pharmacology and Toxicology, University of Zurich, Zurich, Switzerland; 6 Neuroscience Center Zurich, University of Zurich and ETH Zurich, Zurich, Switzerland; 7 INSERM, CNRS, Institut de la Vision, Sorbonne Université, Paris, France; 8 NeuroSpin, Bat. 145, Commissariat à l’Energie Atomique ‘ Saclay Center, Gif-sur-Yvette, France

**Keywords:** Neuro-vascular interactions, Sensory processing

## Abstract

The spatial-temporal sequence of cerebral blood flow (CBF), cerebral blood volume (CBV) and blood velocity changes triggered by neuronal activation is critical for understanding functional brain imaging. This sequence follows a stereotypic pattern of changes across different zones of the vasculature in the olfactory bulb, the first relay of olfaction. However, in the cerebral cortex, where most human brain mapping studies are performed, the timing of activity evoked vascular events remains controversial. Here we utilized a single whisker stimulation model to map out functional hyperemia along vascular arbours from layer II/III to the surface of primary somatosensory cortex, in anesthetized and awake Thy1-GCaMP6 mice. We demonstrate that sensory stimulation triggers an increase in blood velocity within the mid-capillary bed and a dilation of upstream large capillaries, and the penetrating and pial arterioles. We report that under physiological stimulation, response onset times are highly variable across compartments of different vascular arbours. Furthermore, generating transfer functions (TFs) between neuronal Ca^2+^ and vascular dynamics across different brain states demonstrates that anesthesia decelerates neurovascular coupling (NVC). This spatial-temporal pattern of vascular events demonstrates functional diversity not only between different brain regions but also at the level of different vascular arbours within supragranular layers of the cerebral cortex.

## Abstract (web summary)

Rungta et al. utilized single whisker stimulation to map out functional hyperemia along vascular arbours from layer II/III to the surface of primary somatosensory cortex, in anesthetized and awake Thy1-GCaMP6 mice. They demonstrated the presence of a functional diversity not only between different brain regions but also at the level of different vascular arbours within supragranular layers of the cerebral cortex.

## Introduction

Hemodynamic-based imaging techniques such as BOLD and CBV fMRI are commonly used to infer neuronal activation patterns in humans and in animal models. They report dynamic parameters of functional hyperemia, i.e., changes of CBV, CBF, or their consequences on brain oxygenation. However, despite their importance for the interpretation of various functional imaging techniques, the spatial-temporal sequence of these vascular events remains unclear.

Across different brain regions, it has become common practice to name different vessel segments based on their branching order with respect to the upstream arteriole. In the specialized olfactory bulb (OB) glomerular model, synaptic activation is concentrated around the mid-capillary bed, constituted by an extremely dense network of capillaries outlined by long thin-strand pericytes. Upon activation, a vascular signal back propagates rapidly along the vasculature to activate sub-types of mural cells (ensheathing pericytes and smooth muscle cells) on dilating capillaries and arterioles, thereby increasing blood flow in a capillary bed volume larger than that of activated neurons^1^. Importantly, the temporal pattern of diameter changes in OB is stereotypic; the onset of dilation occurs in the parenchymal arteriole and the proximal part of the 1st order emerging branch, and delayed dilation occurring in downstream 2nd to ~4th order capillary branches and upstream pial arteriole. In the rapidly dilating compartment, red blood cell (RBC) velocity can decrease, remain stable, or increase with a delay, and is a poor correlate of blood flow due to a local increase in blood volume^1^. The retina is another region where the spatial-temporal sequence of vascular events has been quantitatively investigated. There, a global light flickering stimulus regulates the amplitude of vessel dilation differently in the superficial, intermediate and deep vascular layers, although, with no statistical difference in onset times between different vascular branch orders^2^. However, whether such stereotyped patterns of activation across specific segments of the vascular arbor extend to higher-order cortical regions remains unclear.

In the cortex, past studies on the dynamics of diameter changes across different types of vessels have yielded inconsistent results^3–7^, some reporting that vasodilation occurs earlier in penetrating arterioles and others in the proximal capillary branches (1st–4th order). One possible explanation underlying these differences is methodological differences between studies; (1) the location of neuronal activation was not systematically mapped out in relation to the vasculature, (2) electrical stimulations were used, (3) the use of acute surgical preparations or anesthetics, (4) the methods used to define the latency of dilation, of importance as baseline vasomotion may vary with the vessel type. In addition to methodological differences, it is important to note that the anatomical and functional properties of vascular compartments could vary beyond the simple distinction based on their vascular branch order. For example, increasing evidence suggests that the “transitional segment”, “pre-capillary arteriole” or “secondary functional unit” (as we named in the OB) must be considered a specific functional compartment, distinct from both the downstream capillary bed and the upstream arteriole. This compartmentalized distinction is highlighted by recent anatomical studies characterizing the presence and variability in the morphology of smooth-muscle cell and pericyte sub-types and their expression of the contractile protein α-SMA, exhibiting variable length and branch order coverage after the penetrating arteriole between different vascular arbors^8–10^, thereby raising the question of whether similar branch order variability could exist in the onset timings of compartment specific vasodilation. Here, we utilized a single whisker deflection paradigm and neuronal Ca^2+^ signals in Thy1-GCaMP6 mice to reinvestigate the degree of stereotypy or diversity of the sequence of diameter and velocity dynamics along layer 2/3 (L2/3) vascular arbors in relation to sensory-evoked neuronal activity.

## Results

**Fig. 1:**
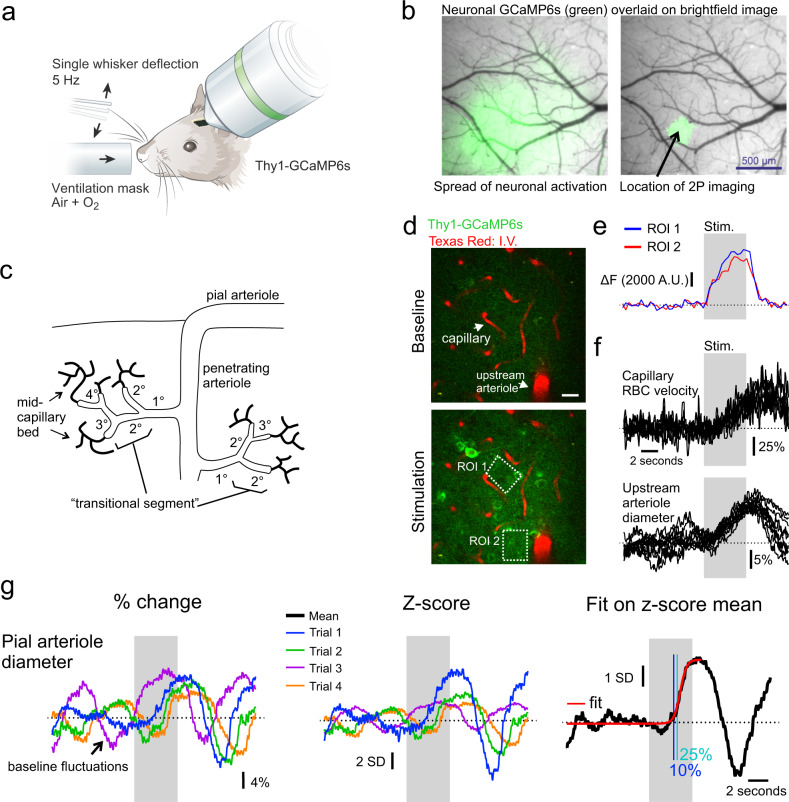
Imaging neurovascular coupling in somatosensory cortex to single whisker stimulations. **a** Schematic of experimental preparation; single whisker of ketamine–medetomidine anesthetized mouse expressing GCaMP6s in pyramidal neurons is deflected at 5 Hz for 5 s. Modified from ref. ^51^. **b** Ca^2+^ signal acquired using epifluorescence and a stereoscope is used to locate area with the largest increase in activity (right). **c** Schematic of vasculature and sites of recordings in L2/3 of vS1. **d** Images showing baseline and stimulation induced increase in neuronal Ca^2+^ surrounding capillaries and upstream arteriole in L2/3. **e** Quantification of Ca^2+^ from 2 ROIs experiment shown in **d**. **f** Increases in capillary velocity (7 trials) and upstream arteriole diameter (11 trials), indicated with arrowheads in **d**. **g** Left, single-trial changes in the diameter of a pial arteriole measured as percentage change from baseline; middle, *z*-score calculation of single trials relative to baseline period; right, average *z*-score of the vessel (black) is fit with a sigmoid (red), the blue line indicates the time at which the fit crosses 10% of peak, and teal line 25% of peak. Gray bar indicates time of stimulation, dotted line indicates the mean baseline value.

We first set out to examine the dynamics of functional hyperemia across the vascular arbor of ketamine–medetomidine anesthetized mice. Chronic glass windows were implanted over vibrissae somatosensory cortex (vS1) of mice expressing GCaMP6s under the Thy1 promoter^11^. All but 1 whisker were trimmed (~0.5 cm) to ensure activation of a single “spared” whisker which was deflected at a rate of 5 Hz (Fig. 1a). In a first step, we used a stereoscope and widefield Ca^2+^ imaging to identify the area of neuronal activation evoked by deflecting the whisker. Consistent with previous studies using voltage indicators^12^, we observed that although only a single whisker was stimulated, the neuronal Ca^2+^ signal spread over an area larger than the barrel column itself (Fig. 1b). Thresholding the signal (see methods) allowed sorting out the area with the largest increase in fluorescence (Fig. 1b) for subsequent 2-photon imaging sessions. Similar to our previous study in OB, we measured functional responses first in capillaries of the mid-capillary bed in relation to local neuronal activity, and then retrogradely traced the direction of blood flow to locate the upstream feeding vessels, i.e., “transitional dilating capillaries”, the 1^st^ order branch, the penetrating arteriole, and the pial arteriole (Fig. 1c), then sequentially measuring the diameter and velocity changes in each compartment. In contrast to the OB, the single whisker stimulation systematically evoked widespread calcium signals in pyramidal cell somata and/or the neuropil, surrounding all types of vascular compartments (Fig. 1d, e), from the mid-capillary bed to the upstream penetrating arteriole. As expected, these calcium responses were followed by increases in red blood cell (RBC) velocity in capillaries and dilations of the upstream arteriole (Fig. 1f). However, in many experiments, the magnitude of the velocity or diameter changes were small compared to the “~0.1 Hz” baseline fluctuations^13,14^. Therefore, to precisely determine and compare the onset time of vessel responses, we computed the *z*-score for each trial with a 9.5 s long baseline (see methods), prior to averaging the trials (Fig. 1g). The data was then fit with a sigmoid function. The time to 25% of the peak of the fit was used to calculate the onset of the diameter change or the RBC velocity change. This systematic approach minimized the contribution of the trials with the largest baseline fluctuations (see a case with particularly large fluctuations in Fig. 1g). Of note, trial-to-trial variability in the magnitude of evoked diameter increases was similar across different compartments with no pattern of adaptation across consecutive trials (Supplementary Fig. 1).

**Fig. 2:**
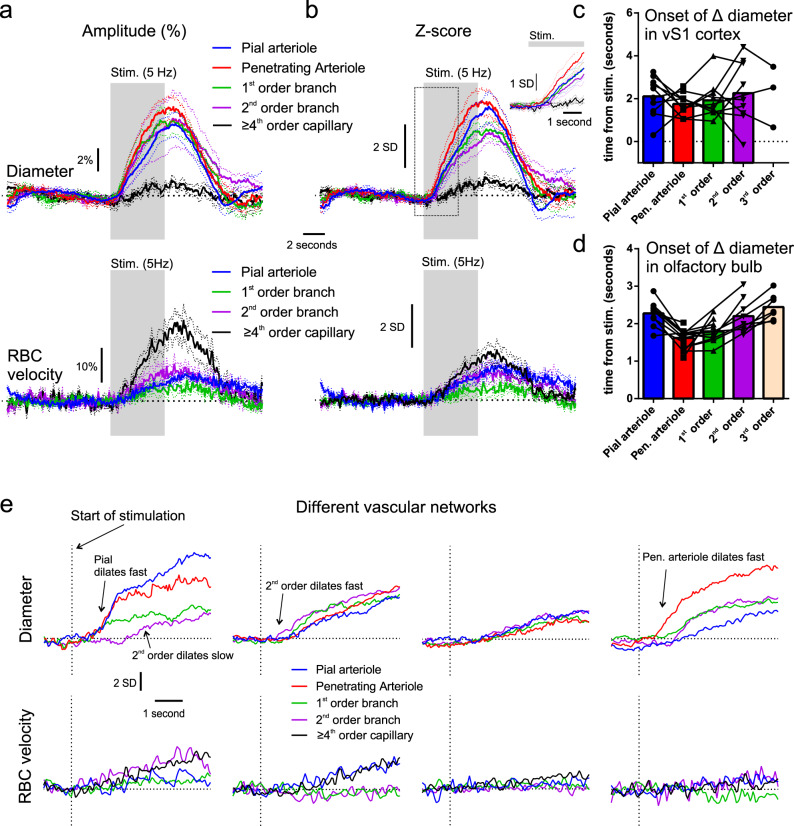
Diversity of functional hyperemia onset times across the vascular arbor of vS1. **a** Average changes in diameter (top) and RBC velocity (bottom) of different vascular compartments in percent increase from baseline. **b** Average *z*-score changes in diameter (top) and RBC velocity (bottom) of different vascular compartments. Dotted lines in **a** and **b** represent SEM. Dotted line box outlines zoomed-in inset in the top right corner. **c** Histogram of onset times (25% of fit peak) across different compartments in vS1 cortex. Data in **a**–**c** represent 10 vascular networks from 6 mice. **d** Histogram of onset times (25% of fit peak) across different compartments in OB, made on a data set primarily formed from previously published experiments^1^. Symbols in **c** and **d** represent data points from individual experiments. **e** Examples of the timing of diameter and velocity increases (*z*-score), from 4 different vascular networks.

Averaged diameter increases across different compartments of 10 vascular networks from 6 mice show that all upstream compartments, from the transitional segment (3rd, 2nd order branches), to the pial arteriole, increase their diameter in response to sensory stimulation (Fig. 2a). However, across the analyzed compartments these dilations were much smaller in percentage change than those previously observed in the OB^1^, (e.g., for penetrating arterioles 8% vs. 29%, for the transitional segment 6% vs. 13%, pial arteriole 6% vs. 27%), and the onset of the diameter increases overlapped from one compartment to another. Note that for higher-order capillaries (≥4th order), diameter changes were minute, as was the case in the OB^1^ and we did not further quantify their onset, although it appeared delayed compared to the local velocity increase in the average across mice. Using the *z*-score approach improved the analysis for individual vessels (Fig. 2b), however, it did not further reveal any statistical differences in timing onsets (Supplementary Data 16, time to 10, 25 and 50% of the peak). Figure 2c, displays the onset times of each compartment across all the vascular networks imaged. Similarly, RBC velocity changes in vS1 were much smaller than those in the OB (Fig. 2a), measured in classical capillaries (≥4th order, lumen diameter of ~2–4 µm) and upstream vessels (vertical penetrating arterioles could not be correctly measured with line scans). This indistinguishable sequence of events in the cortex, contrasts to what was observed in the OB^1^ and resulted from the fact that averaged responses masked a diversity of response onsets along the vascular arbors in the cortex (Fig. 2c) and not in the OB (Fig. 2d). In the OB, the parenchymal arteriole and proximal portion of the 1st order branch dilated faster than downstream 2nd–3rd order vessels and upstream pial arteriole (Fig. 2d, Supplementary Data 16, *z*-score analysis of a data set that is primarily composed of experiments from Rungta et al.^1^). Figure 2e shows examples of timing differences in individual vascular networks in the cortex. In some, the 2nd order vessel dilated fast, whereas in others it dilated with a delay. In others, the pial vessel dilated as fast as the penetrating arteriole, whereas in the OB it was routinely delayed. Finally, velocity responses were also not consistent across cortical vascular arbors and did not always increase in the dilating transitional segment and pial arteriole. Note that the *z*-score responses of velocity were small (Fig. 2b, e), as single-trial responses were not much larger than the resting fluctuations. Overall, the dynamics of functional hyperemia in layer II/III reveal a diversity across vascular networks that contrasts to the OB stereotypy.

We further examined these responses to investigate potential sources of variability. In mouse vS1 the diameter of the first order offshoot from the penetrating arteriole is correlated with α-SMA expression further downstream^9^, raising the question of whether 1st order branch variability could account for some of the variability in onset timing. Consistent with this previously described anatomical correlation, we observed a trend towards faster onset times in second-order vessels originating from large first-order offshoots (>8 µm), compared to those from smaller first-order offshoots (<6 µm) (Supplementary Fig. 2a). We also assessed if baseline vasomotion affected the responses, vasomotion being an indicator of vessel contractility but also spontaneous neuronal oscillations^13,14^. We analyzed the baseline vasomotion by calculating the power density of the low frequency band centered around 0.1 Hz (0.02–1 Hz) for single-trial diameter baselines, and did not detect any correlation between the power density of vasomotion and onset times in any compartments (Supplementary Fig. 2b). However, there was a positive correlation between baseline vasomotion and the amplitude of the diameter increases, which was removed or became slightly negative when plotted against the *z*-score amplitude, reinforcing the use of this approach to normalize responses across trials (Supplementary Fig. 2). Finally, we assessed if the phase of the low frequency vasomotion (~0.1 Hz) across single trials had an effect on the responses as it may be expected that those in a certain phase (i.e., increasing at stim onset), would show stronger responses. No effect of the vasomotion phase on response amplitude was observed across all compartments from the Pia to 2nd order vessels when the responses were normalized to the entire baseline period. However, if alternatively, an offset was applied to co-align the baselines of different phases at stimulation onset, the phase did appear to have an impact on the response as in this case the change from baseline incorporated additive effects of vasomotion and the stimulation evoked response (Supplementary Fig. 3).

**Fig. 3:**
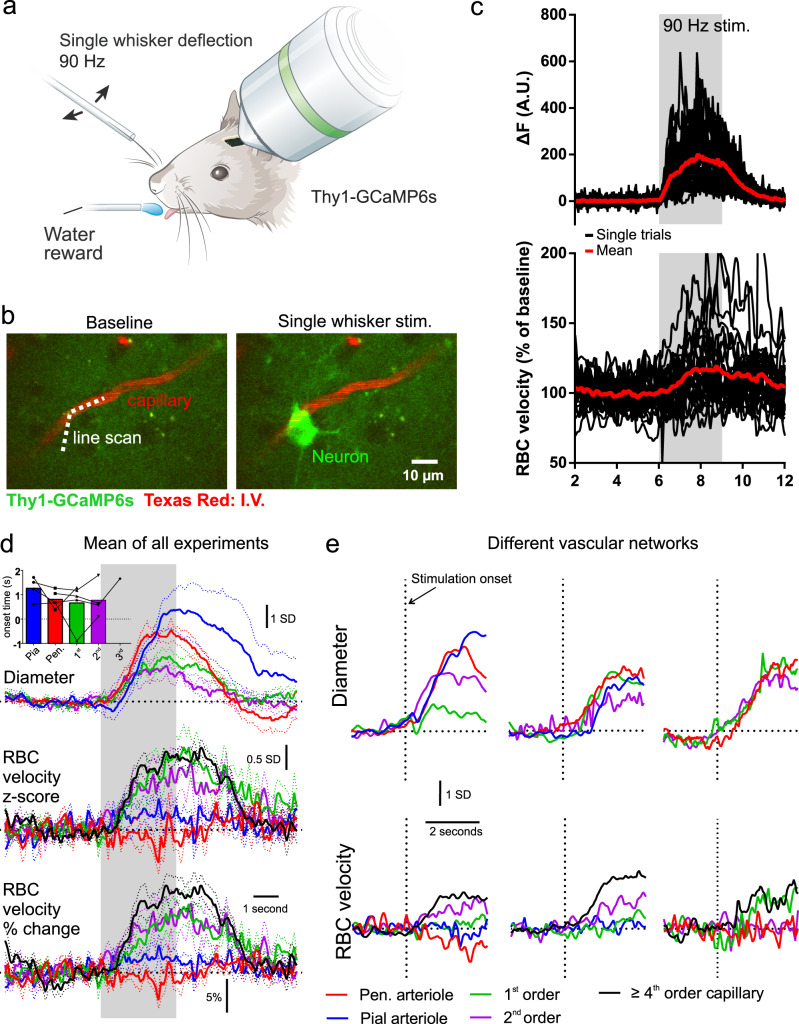
Single whisker evoked vascular dynamics in awake mice. **a** Schematic of experimental setup, single whisker threaded into a capillary tube and deflected at 90 Hz. Modified from ref. ^51^. **b** Example images showing increase in neuronal Ca^2+^ following stimulation adjacent to a capillary labeled with intravenous Texas Red-dextran. **c** Single trials (black) and mean (red) response of neuronal Ca^2+^ (top), and capillary RBC velocity (bottom) to single whisker stim. Data were acquired by scanning along the line path shown in **b** (dotted line) and the portion outside the vessel used to record the Ca^2+^ signal. **d** Average *z*-score changes in diameter (top), RBC velocity (middle) and average % changes in RBC velocity (bottom) across different compartments. Dotted lines represent SEM. Data from 6 whisker/vascular arbor pairs in 4 mice (not all compartments recorded in each network). Inset shows a histogram of onset times (25% of fit peak) across different compartments. **e** Examples of timing of diameter and velocity increases (*z*-score), from 3 different vascular networks.

Next, we aimed to compare these dynamics to the awake mouse (*n* = 6 vascular networks, 4 mice). We used a single whisker stimulation protocol developed for minimizing movements of the mouse during stimulation and which does not evoke increased arousal^15^. The mice were trained to lick a water reward at the end of each trial for >3 weeks prior to recordings, resulting in them staying still during imaging trials. First, we tested whether the single whisker stimulation (90 Hz, 3 s, Fig. 3a), was capable of evoking functional hyperemia in awake mice. Indeed, increases in mid-capillary bed velocity changes were often observed adjacent to neuronal Ca^2+^ increases (Fig. 3b, c), however, both the evoked neuronal and vascular responses under these conditions were variable from one trial to the other, and blood velocity changes were small in amplitude (mean ± SEM, 16.1 ± 1.6 % for ≥4th order capillaries in vS1 cortex, compared to 86.7 ± 5.8% increase in the OB^1^). As in anesthetized mice, the upstream transitional segment, penetrating arteriole, and pial arteriole increased their diameter, however, with variability in diameter and velocity changes across different vascular networks (Fig. 3d, e). Small ≥4th order capillaries remained the compartment of largest RBC velocity increases in percentage, as was observed during anesthesia. In 4 out of 6 vascular networks, we were able to find and image a segment of the penetrating arteriole, which was parallel to the imaging plane for a very brief length, and found that, as expected, RBC velocity did not increase (Fig. 3d), and even appeared to decrease in some cases (Fig. 3e, bottom left), similar to in the OB^1^. Pial arteriole velocity changes also rarely increased under these conditions in awake mice (Fig. 3d, e), suggesting that the increase in flow in arterioles is primarily mediated by a change in blood volume.

**Fig. 4:**
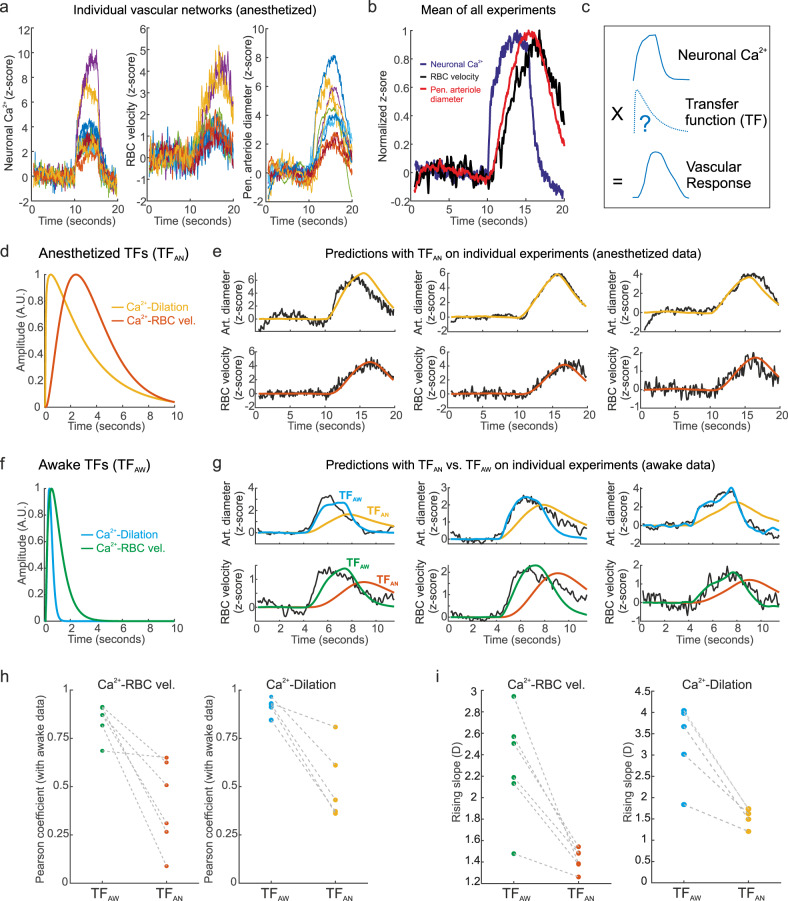
Analysis of differential NVC dynamics in awake vs. anesthetized mice using TFs. **a** Overlaid dynamics of Neuronal Ca^2+^ (GCaMP6 signal), RBC velocity (≥4th order capillaries), and penetrating arteriole diameter from all anesthetized experiments (10 vascular networks, 6 mice). **b** Mean traces of normalized data shown in **a**. **c** Transfer functions (TFs) are convolved with the average neuronal Ca^2+^ signal and optimized to predict the vascular response (RBC velocity or arteriole diameter). **d** TFs optimized to predict arteriole dilation (yellow) or RBC velocity (orange) dynamics computed with the mean anesthetized data. **e** TF predictions for arteriole diameter (top) and RBC velocity (bottom) from the experimental data from 3 different anesthetized experiments (TF_AN_), top and bottom are paired experiments. **f** TFs optimized to predict arteriole dilation (blue) or RBC velocity (green) dynamics computed with the mean awake data (TF_AW_). **g** Comparison of (TF_AW_) and (TF_AN_) prediction for arteriole diameter (top) and RBC velocity (bottom) from the experimental data from 3 different awake experiments, top and bottom are paired experiments. **h** Correlation of (TF_AW_) and (TF_AN_) with awake experimental data for RBC velocity (left) and arteriole diameter (right). **i** Comparison of rising slope for TF predictions made with (TF_AW_) and (TF_AN_) on awake data for RBC velocity (left) and arteriole diameter (right). The slope is defined as the D parameter of the 4-parameter sigmoidal fit formula: *A* + *B*/(1 + exp(−(−x− C) * D)). Data here are also represented in Figs. 1–3.

Finally, we examined the impact of anesthesia on neurovascular coupling (NVC) dynamics. We used transfer functions (TFs) to serve as a proxy for NVC^16^, TFs allowing us to directly compare the relationship between calcium and vascular dynamics, even though the stimulation paradigm differed during brain states (5 s for anesthesia, 3 s for awake). We first computed transfer functions (see ref. ^16^) using the response means across anesthetized mice between neuronal Ca^2+^ and either capillary RBC velocity (Ca^2+^-RBC) or penetrating arteriole dilation (Ca^2+^-dilation) (Fig. 4a–d). Both the Ca^2+^-RBC and the Ca^2+^-dilation TFs predicted their respective signals when tested on experimental data from individual vascular networks of anesthetized mice (Fig. 4e, Pearson coefficient mean ± SD, Ca^2+^-dilation: 0.93 ± 0.04, Ca^2+^-RBC: 0.83 ± 0.11, 10 vascular networks, 6 mice). However, when the same anesthetized TFs (TF_AN_) were used to predict awake responses, they were much less accurate (Fig. 4g, h), with predictions evidently delayed compared to the data. Therefore, we generated new awake TFs (TF_AW_) for both Ca^2+^-dilation and Ca^2+^-RBC, using mean responses of the awake data set (Fig. 4f). Indeed, the TF_AW_ was faster and significantly better than the TF_AN_ at predicting awake data across all networks for both Ca^2+^-dilation and Ca^2+^-RBC (Fig. 4g–i and Supplementary Data 16). These results demonstrate that NVC is faster in awake animals.

## Discussion

Here we address the sequence of compartmentalized vascular changes that occur across vascular networks in L2/3 of primary whisker somatosensory cortex in response to physiological stimulation. Our work extends on previous studies which have examined the dynamics of diameter changes in different compartments in several ways: (1) we trace out and map the vascular network in relation to neuronal activity in transgenic mice, (2) we use a single whisker stimulation to better spatially control the stimulation, (3) our experiments are performed in chronic rather than acute preparations, and were extended to the awake state, (4) we record both diameter and velocity changes across several compartments of the same vascular network from the mid-capillary bed to the upstream pial arteriole, and (5) we investigate the impact of vasomotion on the stimulation driven vasodilation across compartments. Under these conditions, our results reveal that among dilating compartments, the location of onset varies across different vascular arbors. Averaging all responses per compartment masked this diversity and resulted in the inability to identify the fastest dilating compartment. This contrasts with our previous work in the OB, where we observed a more stereotyped pattern of activation. In OB, although intracellular Ca^2+^ dropped synchronously in pericytes of the transitional segment and the upstream arteriole, diameter changes occurred fastest in the parenchymal arteriole and proximal 1st branching vessel^1^.

Although the functional diversity across compartments seems surprising, put in the context of the literature, it is similar to the anatomical diversity which has been described by other groups, in which branch order transition points in the expression of proteins such as smooth-muscle actin can vary across different vascular arbors^8,9,17,18^. Here the purpose of our work was to examine the degree of stereotypy and/or diversity in the timing of compartmentalized functional responses across the vascular arbor, and not add to the controversy over whether capillaries actively dilate which is in part an issue of semantics. It is now evident that different microvascular zones exist^19^, defined by the molecular expression patterns of endothelial and mural cells as well as their morphology^8,9^. Importantly, there exists a transitional segment between the arteriole and mid-capillary bed, in which the mural cells exhibit pericyte-like ovoid cell bodies, and express the contractile protein α-SMA. This transitional segment is analogous to what we called the secondary functional unit in the OB, which actively dilated with a delay. In barrel cortex, the expression of α-SMA is more likely to extend to 2nd and 3rd order branches when the 1st order offshoot of the arbor has a large diameter^9^. Consistent with this anatomical correlation, we observed a trend towards faster dilations of 2nd order vessels arising from larger diameter 1st order offshoots, suggesting that some of the observed functional diversity may be due to branch order variability in α-SMA expression levels. This adds to the emerging picture from several labs showing the importance of the transitional segment in activity-dependent blood-flow regulation^1–5,20–22^. It further highlights an increased level of complexity and the presence of functional diversity between branch orders of this transitional segment. In the mid-capillary bed, thin-strand pericytes do not express or express far lower levels of α-SMA actin^8–10,21^. Although, we remain cautious in interpreting the minute diameter changes observed in high-order capillaries, when averaged across all mice they appeared delayed. Even if passively driven by increased pressure, given their high resistance this would be expected to produce meaningful increases in blood flow^1,23^.

A further complication in determining onset times arises from the spontaneous vasomotion of individual compartments. We found that vasomotion power was significantly correlated with the magnitude of the stimulation evoked diameter changes. Furthermore, at a single trial level, the phase of the low-frequency vasomotion ~0.1 Hz had an impact on the dynamics of the diameter change, indicating that the time-locked response represents a mixture of the stimulation evoked response superimposed upon the vasomotion. To minimize the effect of vasomotion on the onset time calculations we normalized each trial to the standard deviation of the baseline (*z*-score), and then averaged *z*-score trials obtained from each compartment of each arbor. This approach minimized the contribution of those trials with the largest vasomotor fluctuations. We implemented a sigmoid fitting approach and calculated onsets on % peak values of the fit. This removes variability caused by different “signal to noise” levels between different compartments of different arbors, which are subjective to error when using threshold values on the traces themselves (e.g., time to 2 standard deviations). Although we were not able to statistically separate onset times across compartments due to them being masked by variability in the sequence across different arbors, this does not exclude the possibility that differences in mean onset times would become apparent with much larger sample sizes.

The neural circuitry in L2/3 vS1 differs from that of the OB. In the OB, odors selectively activate few glomeruli at low concentration^24,25^. In the mouse, these glomeruli represent individual functional units of ~50–100 µm diameter where an estimated 75,000–150,000 olfactory sensory neuron terminals converge^26,27^ and surround the mid-capillary bed contacted by thin-strand pericytes^1^. In L2/3 of vS1, touch evoked spiking patterns are sparsely distributed^28–31^. As a result, synaptic activity is less spatially concentrated than in OB and occurs more spread out across different compartments of the vascular arbor in vS1. This may contribute to the smaller magnitude vascular responses in vS1 compared to OB and potentially to the greater compartment specific heterogeneity we observed in vS1. vS1 has become the most utilized brain region for the study of neurovascular coupling due to its well-defined circuitry^32^ and vascular anatomy^33^. Here we chose to study the dynamics in superficial L2/3 due to ease of optical access with 2P microscopy and for better comparison with previous studies. In vS1 first order thalamocortical input from VPM arrives predominantly in L4 (and L5b) whereas L2/3 receives second-order projections within a few ms (much faster than the hemodynamic responses that are evoked). To ensure stimulation of a single whisker we trimmed all but one whisker to ~0.5 cm in length, a commonly used protocol which is known to induce circuit remodeling, but importantly in this model, excitatory population responses retain the highest levels of activity within the whisker specific column (L2/3 neurons ~twice as likely to respond to touch as those in neighboring columns^30^). It is important to note that dilation onset times and fMRI responses have been reported to occur faster in deeper layers^7,34^, which may add a further level of complexity to the diversity we report within L2/3. Furthermore, as pial vessel dilation is mediated by the integration of retrograde signals from several penetrating arterioles^35,36^, a possible source of onset variability in the pial arterioles could therefore arise from differences in the arteriole networks they perfuse.

One limitation of this study is that we limit our recordings of neuronal activity to Thy1 expressing excitatory pyramidal neurons. The purpose of recording neuronal activity in this study was to examine the location and timing of the neuronal response, and not intended to shed light on the specific cell types involved. Given the contribution of interneurons and astrocytes to neurovascular coupling^37^, it would indeed be possible for mismatches between the level of local excitatory neuron activity and vascular dilation to occur. Optogenetic stimulation has become a popular tool for dissecting the contribution of specific cell types to increases in blood flow, however, this triggers robust and synchronous activation of populations of cells, which does not replicate their sequence of activation during natural stimulations. As we aim to understand the microvascular changes that contribute to hemodynamic based functional imaging signals, we believe it is important to understand the spatial-temporal pattern of vascular changes to natural stimulations.

It is widely acknowledged that anesthetics affect neurovascular coupling^38–40^. In the OB glomerulus, these effects are likely less dramatic as it is a first synaptic relay and therefore, information is less susceptible to thalamic modulation by anesthesia, in contrast to the neocortex. Using simultaneous measurements of neuronal and vascular responses, we find that in L2/3 the temporal dynamics of functional hyperemia differed according to the brain state. As the onsets and overall dynamics of the neuronal calcium responses in anesthetized and awake animals overlapped, whereas the slope and peak of vascular responses (arteriole dilation and RBC velocity responses) were flatter and delayed during anesthesia, we demonstrate that NVC is faster in awake animals. By computing TFs between calcium and both types of vascular responses, we ensured that this difference was not due to a difference in stimulation duration. These differences in awake vs. anesthetized TFs have important implications for brain mapping studies (e.g., fMRI), which routinely employ hemodynamic response functions to map activity patterns. As the transfer function between neuronal activity and vasodilation is brain state-dependent, modifying the hemodynamic response function to account for differences in brain region and the state of the animal would be important for improving the accuracy of fMRI mapping and interpretation.

In summary, we report functional diversity of neurovascular coupling dynamics between different brain regions (OB vs. vS1 cortex) and between microvascular zones of different vascular arbors within L2/3. These results outline how these different microvascular zones cooperate to increase cerebral blood flow during functional hyperemia in response to a well-defined and physiological sensory stimulation. They also outline the spatial-temporal sequence of blood volume and velocity changes that underlie human brain mapping techniques.

## Methods

### Animals and chronic window implantation

All animal care and experimentation was performed in accordance with the INSERM Animal Care and Use Committee guidelines. Adult mice (2–6 months old, 20–35 g, both males and female, housed in 12-h light-dark cycle) were used in this study. *Thy1-GCaMP6s (GP4.3)* mice were purchased from Jackson laboratory. All mice were bred on a *C57BL/6* background. Chronic craniotomies were performed as previously described^41^. In brief, mice were initially anesthetized with an intraperitoneal (IP) bolus of ketamine–medetomidine (100 and 0.4 mg kg^−1^ body mass, respectively). Further 10–20% of the same mixture was injected IP as necessary to maintain surgical plane anesthesia. During surgery, the mice breathed a mixture of air and supplementary oxygen and the body temperature was monitored with a rectal probe and maintained at ~36.5 °C by a feedback-controlled heating pad. A craniotomy (3.5 mm lateral and 1 mm posterior to bregma) was performed with a dental drill, care was taken not to apply pressure to the bone and the area was regularly flushed with cool aqueous buffer solution to avoid damage or heating of the underlying tissue. A cover glass (100 μm thick) was used for the window and sealed in place with photopolymerizable dental cement, which was also used to form a head-cap in which a head-bar was embedded. Mice were permitted to recover for at least 3 weeks before the imaging sessions began. For anesthetized experiments, mice were anesthetized with ketamine–medetomidine (100 and 0.4 mg kg^−1^ body mass, respectively) injected IP. Experiments were routinely performed between 25 and 120 min after the first injection, and a second bolus IP was occasionally injected during the experiment if the animal’s respiration rate started to increase. Breathing rate (2–3 Hz, regular and rhythmic) was monitored with a pneumogram transducer (Biopac Systems). Body temperature was maintained at ~36.5–37 °C using a heating pad. Blood pressure and heart rate were not measured. Mice breathed a mixture of air and supplementary oxygen (the final inhaled proportion of oxygen was ~30%). Under these same conditions, we have characterized brain temperature at the surface of the brain to be ~32 °C, with capillary PO2 to be similar to in awake mice^42^. No post-mortem analysis was performed to access the integrity of the cortex.

### Imaging

All but one spared whisker were trimmed down to ~0.5–1 cm to facilitate single whisker stimulations. Mapping of epifluorescence GCaMP6 signals was performed on a Zeiss stereomicroscope (Stereo Discovery V20, GFP band pass filter of 525/50). ~5–15 trials were averaged and images were displayed as the integral of the change in fluorescence during the stimulation period relative to the baseline. This mapping procedure was done >3 days before 2-photon imaging sessions began. Signals were thresholded to identify the region with the largest increase in fluorescence and guide subsequent 2-photon imaging experiments. 2-photon imaging was performed as previously described^1^, using a femtosecond laser (Mai Tai eHP; SpectraPhysics) with a dispersion compensation module (Deepsee; Spectraphysics) emitting ~70-fs pulses at 80 MHz. GCaMP6s and Texas Red were excited at 920 nm. Emitted light was collected with either a ×60/1.10NA (Olympus) or ×40/0.8NA (Leica) water immersion objective and was sent to a pair of lenses, coupled into a 2-mm diameter core polymethyl methacrylate optical fiber. Collected light was split using a dichroic mirror at 580 nm and the signals were each detected with a dedicated GaAsP photomultiplier tube (Hamamatsu) after passing through an appropriate emission filter (GCaMP6: 525 nm, 50 nm bp; Texas Red: 620 nm, 60 nm bp). Customized Labview software was used to control imaging parameters. Texas Red dextran (70 kDa, Molecular Probes) was administered intravenously by retro-orbital injection. For awake experiments, mice were briefly (<2 min) anesthetized with isoflurane in order to inject the Texas Red dextran and recovered for >1 h before the experimental session began. Recordings of diameter and velocity across different segments were made sequentially across different trials. In a subset of experiments in which two segments were visible in the same plane, the line scan was extended to capture both of their diameter changes in the same trial.

### Stimulation setups

Awake mouse whisker stimulation was done as previously described^15^. A custom-made head-fixation box was built for chronic imaging and stimulation as previously described in detail^43^. A click noise 1 ms duration with 2–18-kHz bandwidth and delivered by stereo speakers positioned 20 cm away from the animal’s head was sounded to indicate the end of each trial upon which the animal could lick to receive a water reward. The drinking spout included a piezo sensor (LDT0-028K; Measurement Specialties) and was mounted in front of the animal. The spout was connected to a solenoid valve (Type 0330; Burkert) that controlled water delivery upon spout deflection. To stimulate a single whisker, it was threaded into a glass capillary affixed to a piezo element (T223-H4CL-303X; Piezo Systems) vibrated at 90 Hz. A custom-made piezo movement sensor for monitoring movement was positioned under the animal’s body. A camera with an infrared light source was used for monitoring animals. We used the custom-written LabVIEW program (Version 2012; National Instruments) and multifunctional data acquisition cards to control and monitor all components of the behavioral apparatus^43^. For anesthetized mice, the whisker was stimulated with metal rod attached to a mechanical shutter/chopper which deflected the whisker at a rate of 5 Hz.

### Training

Animals were first handled and familiarized with the experimenter, 1 week after implantation at least 2 times a day (~15–20 min) and acclimatized to the behavioral setup. They were gradually accustomed to tolerate brief periods of head fixation and to drink water from a pipette tip administered by the experimenter. Water-deprived mice (12 h before training sessions) were then accustomed to head fixation in the experimental setup and to drink water from a spout delivered at fixed intervals and preceded by a sound cue. Finally, mice were habituated to the stimulation of a random whisker and water delivered at the end of each trial. The total training procedure required a minimum of 3 weeks before data was acquired.

### Transfer function computation

TFs were computed between an input (GCaMP6, Ca^2+^) and an output signal (RBC velocity or arteriolar dilation). Computation was done with a home-made software, based on the scripts from^16,44,45^. The following function was optimized ${\mathrm{TF}}\left(t\right)=H\left(t-{p}_{3}\right){p}_{4}\left(\frac{{\left(t-{p}_{3}\right)}^{{p}_{1}-1}{{p}_{2}}^{{p}_{1}}{e}^{-{p}_{2}\left(t-{p}_{3}\right)}}{\varGamma \left({p}_{1}\right)}\right)$ using the simulated annealing algorithm (“*simulannealbnd*” function, Matlab). Initial values for the parameters were (1.3; 0.5; 0.27; 0.19) as found previously for a TF representing the NVC^16^. Two rounds of 200 optimization runs were performed, the second run initial values being the parameters for the best TF found from the first run. The final TF was the one showing the smallest coefficient of determination between the output signal and the convolution of the TF and the input signal, while being physiologically plausible (i.e., TFs = 0 for time = 0 and none non-derivable point in the TF after the onset).

### Prediction of the TFs computation

Predictions are the result of the convolution between the Ca^2+^ signal of the mouse with the corresponding TF. Amplitude was optimized to match the experimental data when TFs were applied on datasets different from the one on which they have been optimized. The scaling factors were found with the “*fminsearch*” function of Matlab by using the sum of the square residuals as the cost function. The final evaluation of the prediction quality, the Pearson coefficient, is not impacted by the amplitude but only by the dynamic which remains the same with the scale factor.

### Quantification

Ca^2+^ signals were calculated as Δ*F* = (*F − F*_0_) where *F*_0_ represents baseline fluorescence and *F* the fluorescence at time *t* in arbitrary units. Vessel diameter and velocity measurements were made as previously described^1^. Red blood cells were imaged as shadows within the fluorescent plasma and their velocity was calculated based on the distance traveled per unit of time. Lumen diameters were measured with line scans perpendicular to and crossing the vessel and calculated using the fluorescent boundaries of the Texas Red (70 kDa) fluorescence, which labels the blood plasma (excluding the glycocalyx). A 200 ms mean filter (preceding time, *t*) was used, and fluorescence was interpolated between pixels on the distance axis. A subset of the recordings was made in frame scanning mode. *z*-score traces were calculated on individual trials relative to baseline and then averaged across each vessel segment from each vascular network. These average *z*-score traces were fit with a sigmoid, using the method of least squares (Matlab fit function). Onset times were calculated as the time to reach 10, 25, and 50% of the peak on the fit.

### Statistics and reproducibility

The anesthetized data set consisted of data from 10 vascular arbors of 6 mice. The awake data set consisted of 6 vascular arbors from 4 mice. Statistical analysis was conducted using R (version 3.6.2, R Core Team, 2019). Linear mixed-effects models^46^ were used to analyze the differences between onset times of different compartments. The vascular compartment was specified as fixed effects, and intercepts for each mouse were specified as random effects. *P*-values for differences between groups were obtained post-hoc using the Tukey correction for multiple comparisons^47^. All *P*-values are reported in Supplementary data 16. The data from the histograms show data per vascular network, not grouped by the animal. Average data throughout the paper is displayed as the mean ± SEM. No randomization or blinding was used. No statistical methods were used to predetermine sample sizes. A subset of mice did not respond efficiently to the training protocol and were not used to collect data.

### Inter-trial variability

Inter-trial variability was measured from *z*-score traces by calculating the standard deviation (STD) of the response amplitude across trials for each vascular segment from each vascular network. Trial-to-trial adaptation of the response was assessed by comparing the amplitude of the response between the first three trials for each vascular segment.

### Vasomotion estimation

Baseline vasomotion was estimated by performing a power spectrum analysis in the 0.02 to 1 Hz frequency band, encompassing vasomotion oscillations as previously described^48–50^. Average bandpower in the 0.02–1 Hz frequency band was extracted from the baseline of relative diameter change traces (% change) using the classical method (Matlab bandpower function). To analyze the contribution of the low frequency (~0.1 Hz) vasomotion cycle phase on measured dilations, single-trial traces were sorted based on the pattern of vasomotion oscillation before stimulation onset. First, diameter traces relative to the baseline period (% change) were lowpass filtered with a 0.2 Hz threshold. Then, filtered traces were classified based on their oscillation pattern in a 2.5 s window before stimulation onset, corresponding to a quarter of an oscillation cycle at 0.1 Hz. Traces were classified as above baseline average and ascending (phase 1), above baseline average and descending (phase 2), below baseline average and descending (phase 3) or below baseline average and ascending (phase 4) as illustrated in Supplemental Fig. 3A.

### Reporting summary

Further information on research design is available in the Nature Research Reporting Summary linked to this article.

## Supplementary information

Supplemental Material

Description of Supplementary Files

Supplementary Data 1

Supplementary Data 2

Supplementary Data 3

Supplementary Data 4

Supplementary Data 5

Supplementary Data 6

Supplementary Data 7

Supplementary Data 8

Supplementary Data 9

Supplementary Data 10

Supplementary Data 11

Supplementary Data 12

Supplementary Data 13

Supplementary Data 14

Supplementary Data 15

Supplementary Data 16

Reporting Summary

## Data Availability

Data files associated with the manuscript are available as Supplementary Data 1–15.

## Code availability

Code and software used to generate transfer functions are available online^44,45^ 10.5281/zenodo.4765555.
