# Supplementary material for: Diversity of neurovascular coupling dynamics along vascular arbors in layer II/III somatosensory cortex
Source: Commun Biol. 2021 Jul 9;4:855. doi: 10.1038/s42003-021-02382-w (PMC8270975; doi:10.1038/s42003-021-02382-w)
Supplement: Supplementary file 1 — Supplemental Material [file 42003_2021_2382_MOESM1_ESM.pdf]

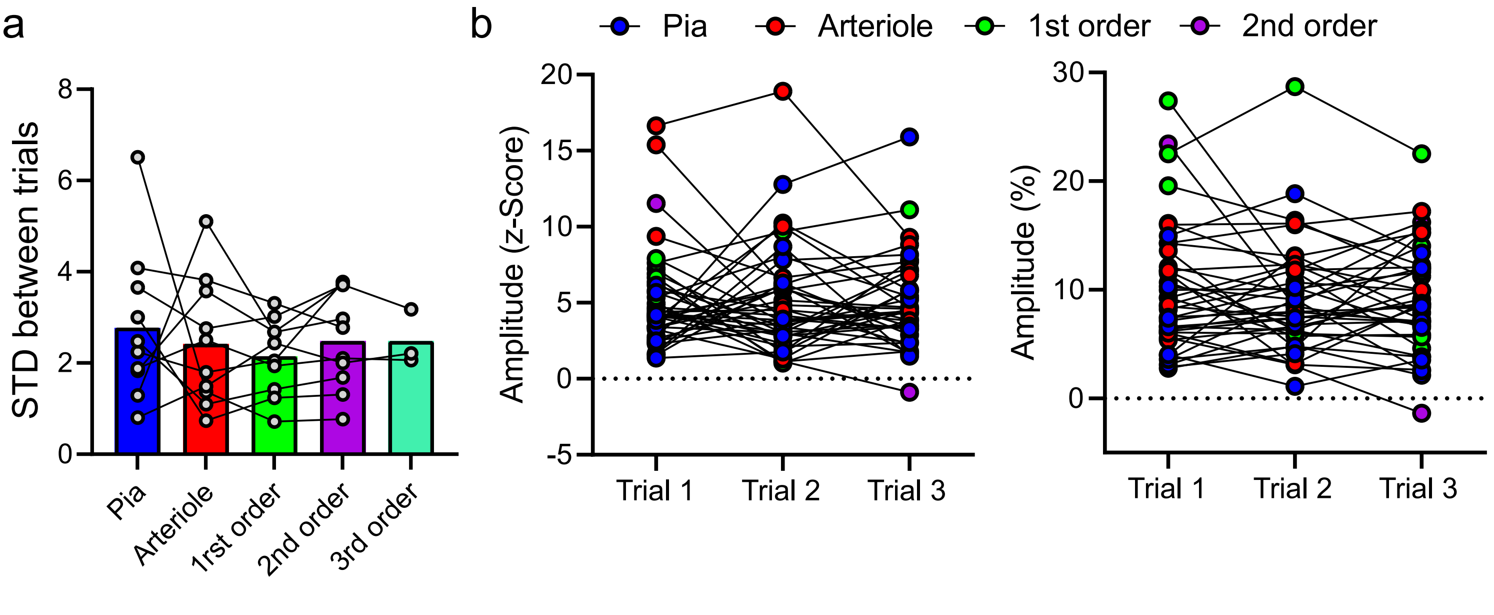

Supplementary Figure 1: Individual trial variability

a) STD of z-score peak amplitude between different trials of individual vascular networks (connected lines), sorted by compartment.
b) No pattern of trial-to-trial adaptation was observed across consecutive trials. Left, z-score peak; Right, percent increase from baseline.

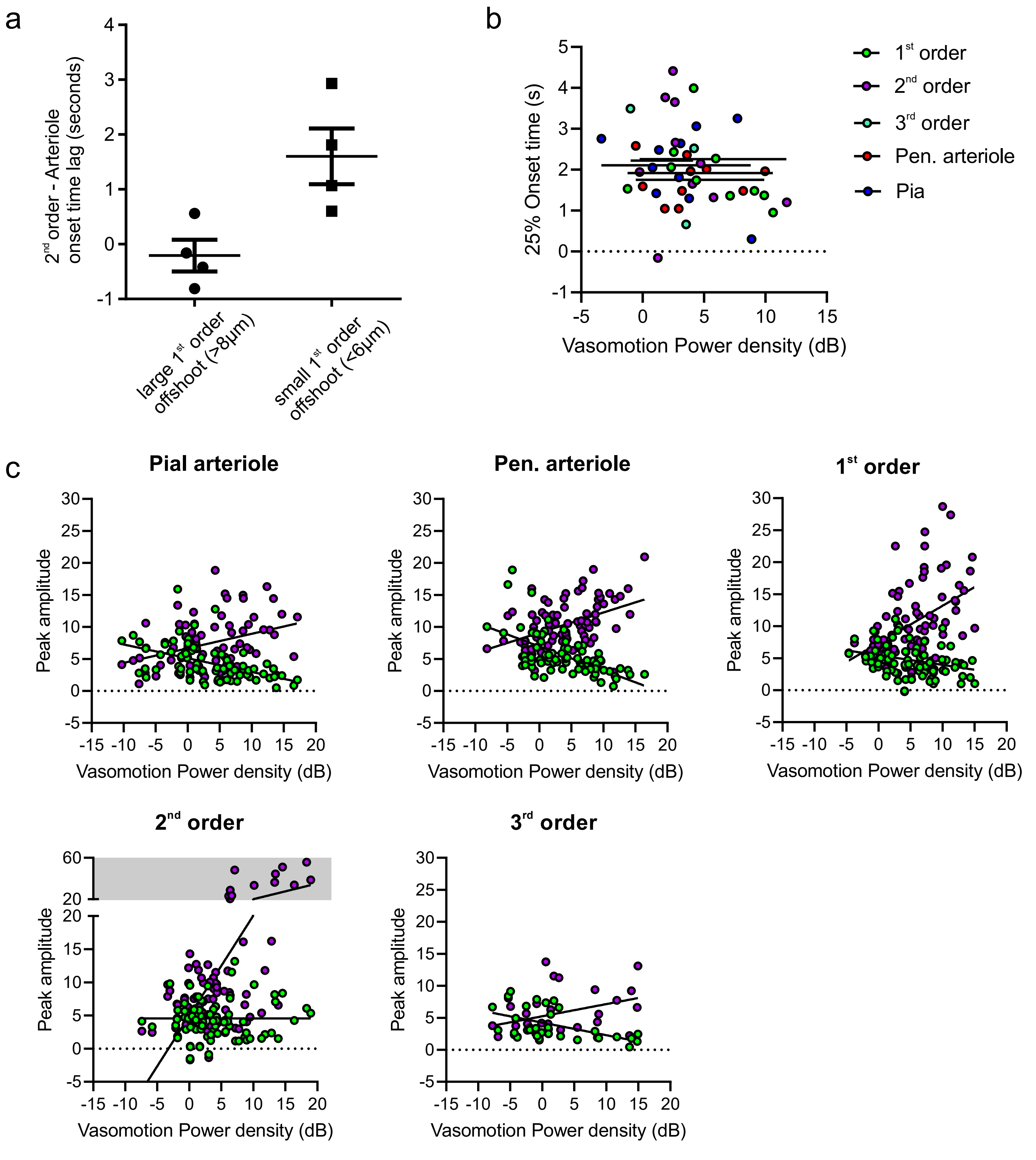

See legend on next page.

Supplementary Figure 2: Relationship between first order offshoot size and vasomotion with stimulation evoked diameter increases.

a) Difference in onset times between connected arteriole – 2nd order branch pairs (lag), on vascular arbours with large vs. small 1st order branch offshoots. $p = 0.01$, paired t-test if different vascular networks from same mouse considered independent, $p = 0.06$ when grouped by animal (see methods), $n = 8$ vascular networks, 6 mice.
b) Onset time is not correlated with vasomotion power, colour coded circles represent trial averaged data from individual vascular arbours. None of the fits significantly deviated from 0.
c) The stimulation evoked diameter peak amplitude (% change, purple) and (z-score, green) vs. vasomotion power. Data points are single trial data across multiple vascular arbours. Shaded area on 2nd order plot represents a re-scaling of the y-axis. All fits of data in % change relative to baseline (purple) significantly deviated from 0 ($p < 0.05$).

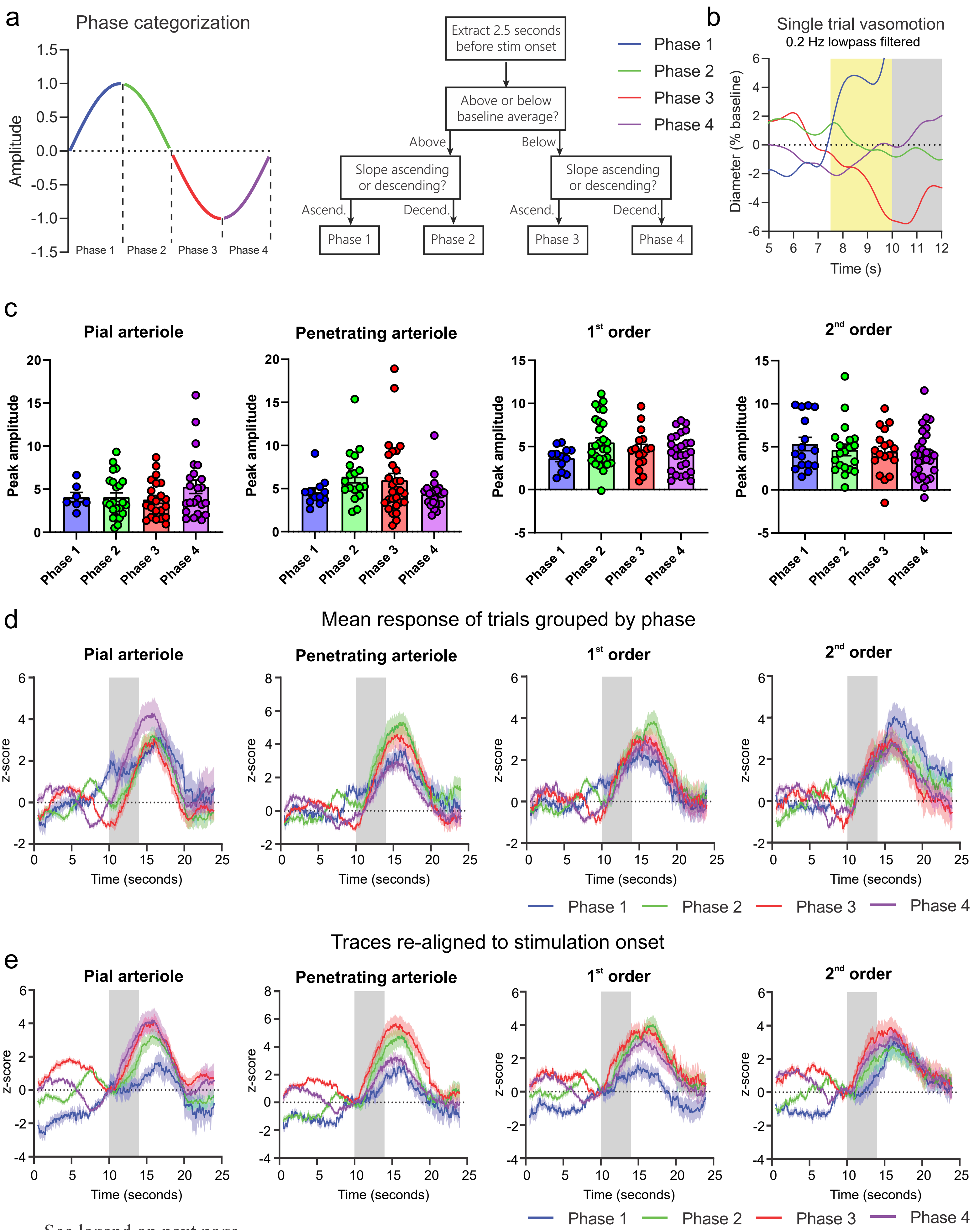

See legend on next page.

Supplementary Figure 3: Relationship between low frequency vasomotion phase and stimulation evoked response.

a) Flow chart describing single trial phase classification as "Phase 1-4"
b) Four trials from a pial arteriole lowpass filtered and characterized as different phases by analyzing the polarity and direction in the yellow shaded area (2.5 seconds).
c) Peak amplitude of each trial grouped by their phase classification for different vascular segments.
d) Average traces obtained from trials grouped according to phase.
e) Same as in (d), except the baseline was re-aligned to the time of stimulation onset (10 seconds). Shaded colours in (d and e) represents standard error of the mean (SEM). Grey bar indicates time of whisker stimulation.
