# Supplementary material for: Diversity of neurovascular coupling dynamics along vascular arbors in layer II/III somatosensory cortex
Source: Commun Biol. 2021 Jul 9;4:855. doi: 10.1038/s42003-021-02382-w (PMC8270975; doi:10.1038/s42003-021-02382-w)
Supplement: Supplementary file 2 — Description of Supplementary Files [file 42003_2021_2382_MOESM2_ESM.pdf]

Description of Additional Supplementary Files

File name: Supplementary Data 1
Description: Source data Figure 1e.

File name: Supplementary Data 2
Description: Source data Figure 1f.

File name: Supplementary Data 3
Description: Source data Figure 1g.

File name: Supplementary Data 4
Description: Source data Figure 2a; diameter.

File name: Supplementary Data 5
Description: Source data Figure 2; velocity.

File name: Supplementary Data 6
Description: Source data Figures 2b, e; diameter.

File name: Supplementary Data 7
Description: Source data Figure 2b, e; velocity.

File name: Supplementary Data 8
Description: Source data Figure 2c, onsets cortex.

File name: Supplementary Data 9
Description: Source data Figure 2d, onsets OB.

File name: Supplementary Data 10
Description: Source data Figure 3d, diameter.

File name: Supplementary Data 11
Description: Source data from Figure 3d, velocity percent.

File name: Supplementary Data 12
Description: Source data from Figure 3d, velocity z-score.

File name: Supplementary Data 13
Description: Source data from Figure 3d, onsets.

File name: Supplementary Data 14

Description: Source data Figure 4 a, b; diameter and velocity data from Supplementary Data files 5 and 6.

File name: Supplementary Data 15
Description: Source data related to Figure 4 e, h, i.

File name: Supplementary Data 16
Description: Supplementary Table 1 lists p-values associated with data and statistical tests throughout the manuscript and figures.
