# Supplementary material for: Diversity of neurovascular coupling dynamics along vascular arbors in layer II/III somatosensory cortex
Source: Commun Biol. 2021 Jul 9;4:855. doi: 10.1038/s42003-021-02382-w (PMC8270975; doi:10.1038/s42003-021-02382-w)
Supplement: Supplementary file 19 — Reporting Summary [file 42003_2021_2382_MOESM19_ESM.pdf]

nature research

Corresponding author(s): Ravi Rungta, Serge Charpak

Last updated by author(s): May 12, 2021

Reporting Summary

Nature Research wishes to improve the reproducibility of the work that we publish. This form provides structure for consistency and transparency in reporting. For further information on Nature Research policies, see our Editorial Policies and the Editorial Policy Checklist.

Statistics

For all statistical analyses, confirm that the following items are present in the figure legend, table legend, main text, or Methods section.

n/a	Confirmed	
☐	☒	The exact sample size (*n*) for each experimental group/condition, given as a discrete number and unit of measurement
☐	☒	A statement on whether measurements were taken from distinct samples or whether the same sample was measured repeatedly
☐	☒	The statistical test(s) used AND whether they are one- or two-sided<br>*Only common tests should be described solely by name; describe more complex techniques in the Methods section.*
☒	☐	A description of all covariates tested
☐	☒	A description of any assumptions or corrections, such as tests of normality and adjustment for multiple comparisons
☐	☒	A full description of the statistical parameters including central tendency (e.g. means) or other basic estimates (e.g. regression coefficient) AND variation (e.g. standard deviation) or associated estimates of uncertainty (e.g. confidence intervals)
☐	☒	For null hypothesis testing, the test statistic (e.g. *F*, *t*, *r*) with confidence intervals, effect sizes, degrees of freedom and *P* value noted<br>*Give P values as exact values whenever suitable.*
☒	☐	For Bayesian analysis, information on the choice of priors and Markov chain Monte Carlo settings
☒	☐	For hierarchical and complex designs, identification of the appropriate level for tests and full reporting of outcomes
☒	☐	Estimates of effect sizes (e.g. Cohen's *d*, Pearson's *r*), indicating how they were calculated

Our web collection on statistics for biologists contains articles on many of the points above.

Software and code

Policy information about availability of computer code

Data collection	Data was collected using custom labview based software.
Data analysis	Data was analyzed using custom labview and matlab software. Software for transfer functions can be found available in reference 43.

For manuscripts utilizing custom algorithms or software that are central to the research but not yet described in published literature, software must be made available to editors and reviewers. We strongly encourage code deposition in a community repository (e.g. GitHub). See the Nature Research guidelines for submitting code & software for further information.

Data

Policy information about availability of data

All manuscripts must include a data availability statement. This statement should provide the following information, where applicable:

- Accession codes, unique identifiers, or web links for publicly available datasets
- A list of figures that have associated raw data
- A description of any restrictions on data availability

The source data associated with the main figures is available as supplementary data files 1-15

Field-specific reporting

Life sciences study design

All studies must disclose on these points even when the disclosure is negative.

Sample size	Sample sizes were chosen to be consistent with previous studies
Data exclusions	Data was not analyzed if movement caused the vessel of interest to move in the z-plane.
Replication	Multiple trials were conducted per vascular segment. The anesthetized data set consisted of data from 10 vascular arbors of 6 mice. The awake data set consisted of 6 vascular arbours from 4 mice.
Randomization	No randomization was performed
Blinding	No experimental treatments were compared, and no blinding was performed

Reporting for specific materials, systems and methods

We require information from authors about some types of materials, experimental systems and methods used in many studies. Here, indicate whether each material, system or method listed is relevant to your study. If you are not sure if a list item applies to your research, read the appropriate section before selecting a response.

Materials & experimental systems

n/a	Involved in the study	
☒	☐	Antibodies
☒	☐	Eukaryotic cell lines
☒	☐	Palaeontology and archaeology
☐	☒	Animals and other organisms
☒	☐	Human research participants
☒	☐	Clinical data
☒	☐	Dual use research of concern

Methods

n/a	Involved in the study	
☒	☐	ChIP-seq
☒	☐	Flow cytometry
☒	☐	MRI-based neuroimaging

Animals and other organisms

Policy information about studies involving animals; ARRIVE guidelines recommended for reporting animal research

Laboratory animals	Adult mice (2–6 months old, 20–35 g, both males and female, housed in 12-hr light-dark cycle) were used in this study. Thy1-GCaMP6s (GP4.3) mice were purchased from Jackson laboratory. All mice were bred on a C57BL/6 background.
Wild animals	N/A
Field-collected samples	N/A
Ethics oversight	All animal care and experimentation was performed in accordance with the INSERM Animal Care and Use Committee guidelines.

Note that full information on the approval of the study protocol must also be provided in the manuscript.
